# Multi-level and hybrid modelling approaches for systems biology

**DOI:** 10.1016/j.csbj.2017.07.005

**Published:** 2017-08-10

**Authors:** R. Bardini, G. Politano, A. Benso, S. Di Carlo

**Affiliations:** Politecnico di Torino, Department of Control and Computer Engineering, 10129 Torino, Italy

**Keywords:** Systems biology, Computational biology, Multi-level models, Hybrid models, -omics, Multi-scale systems

## Abstract

During the last decades, high-throughput techniques allowed for the extraction of a huge amount of data from biological systems, unveiling more of their underling complexity. Biological systems encompass a wide range of space and time scales, functioning according to flexible hierarchies of mechanisms making an intertwined and dynamic interplay of regulations. This becomes particularly evident in processes such as ontogenesis, where regulative assets change according to process context and timing, making structural phenotype and architectural complexities emerge from a single cell, through local interactions. The information collected from biological systems are naturally organized according to the functional levels composing the system itself. In systems biology, biological information often comes from overlapping but different scientific domains, each one having its own way of representing phenomena under study. That is, the different parts of the system to be modelled may be described with different formalisms. For a model to have improved accuracy and capability for making a good knowledge base, it is good to comprise different system levels, suitably handling the relative formalisms. Models which are both multi-level and hybrid satisfy both these requirements, making a very useful tool in computational systems biology. This paper reviews some of the main contributions in this field.

## 1 Introduction

Systems biology considers biological entities as complex holistic structures whose behaviour cannot be reduced to the linear sum of the functions of their parts [1]. With the aim of gaining a deeper insight over biological complexity, computational modelling and simulation can support the understanding of experimental data, as well as the capability of generating and testing hypotheses about them [2]. However, given the huge complexity and peculiar features of these systems, it is necessary to carefully understand the specific modelling requirements they pose, in order to define what a good model for systems biology should look like.

In a complex biological structure, overall features emerge from local interactions among its sub-parts [3]. These interactions are in general favoured by the spatial proximity of the sub-parts. *Spatiality* is therefore one of the biological characteristics that must be taken into account when modelling biological systems [4]. More specifically, the probability of two elements to interact is a function of their spatial proximity and the stochasticity guiding such events must be explicitly taken into account in the modelling task [5].

Biological systems evolved different strategies to control the probability of interaction between biological components. One of them is called *compartmentalization* [6], [7]. Biological systems are organized in compartments, and boundaries between compartments selectively regulate the passage of molecules, thus altering the probability density over space of molecular encounters. In a model, this must translate into the capability of expressing encapsulation and selective communication of each sub-part [8].

Spatial proximity between molecules not always translates into functional activations. The activation of selected functions, in fact, may require biochemical interactions between the molecules leading to structural changes able to alter their functional state. Structural features of biomolecules are encoded in the genome. Thus, the way such information is used determines the quality and quantity of actors and their interactions. The usage of genomic information is regulated at different levels and by different mechanisms, which are in flexible hierarchical relations. Such dynamic interplay of regulations is made of hierarchic relative relations that change according to the process context. This corresponds to the definition of epigenetic regulation in its broader sense: everything acting between a genotype and the corresponding possible phenotypes [9]. Biological models therefore require efficient ways to represent context-dependent and flexible hierarchies.

The modelling of biological systems should also comprise their quantitative aspects. Nevertheless, the way this is taken into account depends on the context. Some biological phenomena fit better with qualitative and discrete information. In other cases, biological quantities need to be represented with continuous quantities, for example referring to molecular concentrations. Therefore, a good model must be able to handle discrete and continuous variables as well as qualitative and quantitative information.

In the large variety of problems to be tackled with a systems biology modelling approach, ontogenetic processes are an example of how the presented modelling requirements are pushed to an extreme. Ontogeny takes the individual organism from the stage of fertilized egg to its fully developed form [10]. This involves a finely tuned and context-dependent processing of the spatiotemporal regulation of the genomic information. In fact, (almost) all cells in an organism share the same genome, yet they have different functional specializations and the overall system exhibits architectural and phenotype diversity. During development, cells undergo differentiation processes guided by their internal states as well as by extrinsic signals. Such signals come from other cells, which are in turn undergoing the same kind of regulations. These inter-cellular interactions can be mediated by concentration gradients over space: different relative positions between the sender and the receiver correspond to different concentration levels determining different results for the same signal. Depending on the context of the process (cellular micro-environment, developmental phase, cell types under analysis, specific regulative state of the cell, etc.) the different regulatory mechanisms involved in ontogenesis change their relative hierarchical relations. In turn, this means that sometimes the genetic regulation determines the future epigenetic state of the cell, other times it is the epigenetic state that determines the availability of the genomic information required to trigger the genetic regulations.

## 2 An introduction to hybrid and multi-level models

As discussed in the introduction, systems biology models in general must be able to handle different scales of representation, to model the system and its sub-parts into a complex hierarchical structure and to handle various types of information represented with different formalisms.

This review focuses on a particular class of models usually referred to as multi-level and hybrid models. Multi-level models describe a system at least at two different levels. Interactions are taking place within and between those levels [11]. Multi-level models allow for the explicit representation of “upward” and “downward” relations. Upward relations model the fact that the system is somehow constrained by the behaviour of its parts, but at the same time downward relations model the fact that the behaviour of each part is influenced by the behaviour of the system as a whole.

When considering multi-level models it is important to make an explicit distinction between the concept of scale and the concept of level [12]. More specifically, the concept of scale refers to a measurable dimension of the analysis of the considered phenomenon. This dimension can be spatial, temporal, and quantitative. The spatial dimension refers to the size of the entities involved in the phenomenon whereas the temporal dimension is related to the timing associated with the behaviours of these entities and their interactions. The quantitative dimension instead refers to the amount of entities involved in the phenomenon. Differently, the concept of level provides a way to locate the studied phenomenon and/or the entities involved in a phenomenon along the considered dimension of the analysis. A level usually corresponds to all the entities whose size and/or characteristic evolution time have the same or comparable orders of magnitude. For example, a system could be represented at the atomic, molecular, cell, organ, population level.

The concept of multi-level models can be coupled with the concept of hybrid models. According to Stephanou et al., “in its most general definition, a hybrid model corresponds to any interaction or coupling between two or more models that are not based on the same formalism” [13].

Based on this definition, we define models which are both multi-level and hybrid as representations supporting different formalisms and organized in levels encompassing multiple systems scales.

**Fig. 1:**
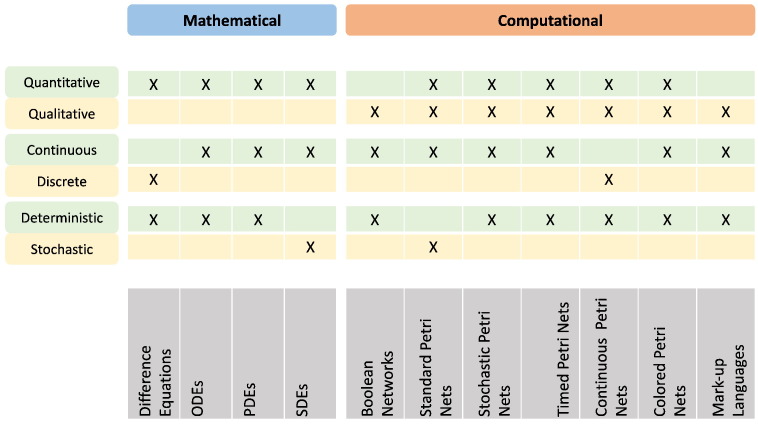
Formalisms employed in modelling systems biology and their main features.

When building up a multi-level and hybrid model, besides choosing the interesting organizational levels, it is necessary to choose the formalisms to describe the different components in the overall model structure. In this sense, it can be useful to briefly revise the formalisms more often employed in modelling biological systems, so that their strengths and limitations can be taken into account when selecting hybrid combinations for the different organizational levels to be modelled. Fig. 1 summarizes the set of considered formalisms and their main characteristics. For a more detailed review of the modelling formalisms used in systems biology, see [14].

In general, biological systems models can be distinguished into mathematical and computational ones. “A computational model is a formal model whose primary semantics is operational; that is, the model prescribes a sequence of steps or instructions that can be executed by an abstract machine, which can be implemented on a real computer. A mathematical model is a formal model whose primary semantics is denotational; that is, the model describes by equations a relationship between quantities and how they change over time.” [15] However, this separation is not strict. Mathematical models can be simulated as well, with the only difference that the computational effort lies into the algorithm chosen to solve the model. One can get insights from a computational model by executing it, or by analyzing it by means of tools for model checking. Mathematical models can instead provide information through formal analysis, but they can be also simulated and solved.

Both mathematical and computational formalisms can be then categorized according to similar opposite features: they can be either qualitative or quantitative, discrete or continuous, deterministic or stochastic.

Usually, mathematical models are based on systems of equations. Difference equations are one of the preferred formalisms when modelling the system using discrete terms [16]. Instead, differential equations are among the preferred formalisms if the model is based on a representation of continuous biological quantities. Ordinary Differential Equations (ODE) are in general used whenever only the temporal aspects of the system are taken into account. Partial Differential Equations (PDE) can instead be used when modelling spatial variations, while Stochastic Differential Equations (SDE) are better suited when considering stochasticity in the model. Such sets of equations can be simulated to study the system evolution and the equilibrium properties [17], [18].

Biological computational models can benefit from a variety of existing approaches and tools. Models can be based on boolean networks, Petri nets, interacting state machines, process algebra, rule-based systems or state charts. Spatiality can be included in the system representation with spatio-temporal models, which can be compartment-, agent- or lattice-based [15], [19].

## 3 Review of existing approaches for multi-level and hybrid models

This section reviews some of the most relevant multi-level and hybrid modelling approaches presented in the literature. The review is organized presenting the different models based on their relevance to the different aspects of the modelling process.

### 3.1 Data collection, organization, integration

One of the motivations for multi-level and hybrid models approaches stems from data availability. High-throughput technologies make possible to extract large quantities of heterogenous data from different organizational levels in biological systems [20].

The study of living systems can produce a variety of different data. To better describe the system of interest, heterogenous data must be included in the model. For example, by combining biological and physiological information it is possible to gain a better insight over a biological system. The first step towards integrating different kinds of information in a model is to collect the raw data required to build the information. For example, in the context of the Physiome Project, the insilicoDB collects physiological experimental data (time-series and image-based morphological models) [21]. Information in the database can be used together with modules representing biological mechanisms to build up and simulate multi-scale and multi-level models of human physiology.

Another example of systematic data aggregation is proposed in [22], where the potential of a multi-level approach to the study of breast cancer relies on the integration of molecular information and system-level functional descriptions. The platform, which is centred on data integration, also allows for queries in existing ontologies, and to perform analyses and modelling efforts over the stratified data.

Other integrative approaches can be used when dealing with different -omic data from different levels of organization in the same system. In fact the data analysis task for extracting information from the complete exploration of genomic, epigenomic, transcriptomic, translatomic, proteomic, metabolomic, and phenomic data of a system is not trivial.

In [23], the authors revise a set of integrative inference and analysis techniques for omics data sets generated from different cellular levels. The considered approaches are mainly based on the analysis of associations and correlations between two levels, and on co-regulation studies. Also, according to [23], time resolved experiments can be modelled with time-series analysis of how a system perturbation spreads from one level to another. Such approach can be extended to population of organisms adapting to different environmental conditions affecting their regulative state. What emerges from the literature is that tools for data integration are often suited for only two data sources. However, when considering systems with multiple levels and different data types, dedicated analysis methods must be used and developed.

Sometimes, it is not possible to obtain the data of interest for a phenomenon under study. For instance, kinetic parameters of metabolic reactions are limited to equilibrium states, only. Also, experimental data often refer to *in vitro* studies, and not to the *in vivo* system where different conditions affect the parameters under study. This lack of data is due to technological limitations that probably will not change in the near future, thus creating the need to develop alternative strategies for studying the related phenomena. Dealing with limitations in data availability is not trivial; Subsection 3.3 will discuss some of the related problems and interested readers can refer to [24] for a review of modelling approaches dealing with limitations in data availability.

### 3.2 Model construction and composition

The model construction starts from the available data and leads to the creation of the model. This can happen starting from a deductive, hypothesis-based process as well as from an inductive, data-driven process [15], [25], [26].

Multi-level and hybrid models often take shape from the composition of existing models [27]. Such models are usually already complete and validated, but composing them introduces the need to test the resulting overall model for consistency. Therefore, the choice of the proper formalism and of suitable correspondences between the system and the parts of the model becomes somehow secondary to the task of integrating existing models, which already solved specific issues in independent and different ways.

Multi-level models often deal with two levels of organization: a micro and a macro level, and relations between the two levels can be described as upward or downward causations [28]. A number of strategies do exist for representing how sub-parts of a system at the micro-level do influence the system as a whole at the macro-level, and how the system as a whole does influence its parts [29].

DEVS (Discrete Event Systems Specification) is a formalism supporting this modelling strategy. In its original formulation it includes coupled modules (at the macro level) acting as mere executives for atomic models representing the parts of the system (at the micro level) [30]. One of its main drawbacks is that it is not possible to set global variables affecting the behaviour of sub-models, and all interactions at the micro level happen asynchronously. In [28], the authors present a multi-level-DEVS formalism which overcomes these limitations in two ways: (1) the coupled model has a state and a behaviour of its own, and (2) the upward and downward exchanges between levels are explicitly defined through a system of ports allowing for selective communication. Moreover, discrete state changes at the macro level can emerge from threshold crossings at the micro level. The macro level in turn can activate modules at the micro level sending them events. Finally, downward and upward activations are synchronous.

A typical application of this multi-level model construction is the study of tumor growth. Tumor growth is a biological phenomenon studied with different independent strategies and scopes. On one side, a growing tumor can be considered at the macro scale, as a single entity inside an organism. On the other side, at the micro level it can be considered as a complex structure whose behaviour emerges from local interactions between single cells. It is also possible to get an insight over tumor growth considering a meso level, where the significant entities are aggregates of cells interacting with their environment [31]. In [32], the authors describe a multi-level model of *in vitro* tumor spheroids and the effects of environmental stimuli on their growth. Cellular aggregates make the lower level in the model, while the macroscopic regulations make the higher one. The major contribution of this work is the construction of an intermediate model interfacing the relative two models. Such structure is able to put in the correct relations input and output functions between the levels, making them communicating in a way which is consistent with the experimental data to be modelled. Since the two bridged models stem from independent model construction processes, the fact that they can generate consistent behaviors makes a sort of mutual validation for both of them. This highlights one of the advantages hybrid modelling strategies could provide: inherent validation by direct comparison of independently developed models to be combined [32].

In [33] the authors propose a versatile platform for integrating two modelling mark-up languages. This strategy leverages the modelling power, usability and interoperability of two language-based existing approaches named systems biology markup language (SMBL) and Physiological Hierarchy Markup Language (PHML), combining the respective advantages of the two languages. SBML [34] is better suited for representing sub-cellular mechanisms in an ODE-based way. PHML is dedicated to the representation of hierarchically organized systems, being the successor of *insilicoML* [35]. SBML modules are embedded into a PHML framework, resulting in the capability of accurately representing several organizational levels of a system in the same model. When binding different modules, input and output functions must be put in proper relation. That happens with functions which “get” or “set” values from or into other module variables. This approach introduces the need to verify afterwards the consistency of the resulting model. A model structured this way can become computationally expensive from the simulation perspective. Issues related to the simulation of the models will be discussed in Section 4.

Most often, multi-level modelling approaches to systems biology deal with multi-cellular systems. More rarely, they face the challenge of modelling a whole organ. This is the case of the virtual liver, a project inspired by the previous efforts for building up a virtual heart in the context of the Physiome Project [36]. The virtual liver encompasses a wide range of different time and space scales: from seconds required to a hormone to exert its action on cellular receptors to weeks taken by tissue regeneration; from the micro scale of single cell systems to the scale of the whole organism containing the modelled organ. In fact, one of the modules composing the model refers to the whole body, in this case represented with a Physiologically Based Pharmacokinetic (PBPK) model [37], considering the contribution of all body districts to the context in which the liver works. This approach includes a variety of functional levels: a Perfusion module deals with incoming blood flow at the organ level. Under the assumption of micro-homogeneity, the model takes into account anatomical architecture at the organ level, but considers the smallest functional units to share homogeneous structures. Those are Lobules, coming with sets of cells being the smallest units able to recapitulate the organ function. These homogeneous liver units seem to function at a steady state most times, since cell replacement occurs at a very slow time scale than that of other modelled processes. Still, sometimes, like in the case of tissue regeneration, cell number and identity change at a faster time scale, and single cells affect process evolution. Cells are then represented in an agent-based way, so their specific reactions to environmental changes can emerge in a realistic way. All modules in the model are coupled: in principle, every slight change of a variable in a module could affect the entire system. Given the huge quantity of processes taken into account and the fact that the model uses different mathematical formalisms for the modules and their interactions, simulation faces a huge computational complexity.

### 3.3 Parametrization and parameter identification

As a broad definition, parametrization is the representation of a physical effect by using simplified parameters in a model rather than by computing them dynamically [38]. When focusing on mathematical models, this problem becomes particularly critical because it requires to find a set of parametric equations describing the system.

Parameters are numerical or other measurable factors that define specific aspects of the system.

In some systems, like for example in modelling Newton's laws, these parameters (the force due to gravity and the masses of the objects) are known. Unfortunately, in most biological systems some or many of the parameters are either unknown or significantly uncertain. They often represent phenomena too small or too complex to be treated as system variables or even measured. In this case, parameters are said to be loosely constrained, or ill conditioned [39]. The parameters of a system set the degrees of freedom of the system itself and their identification is the task of estimating their value for a given model. Parameter values are usually estimated by fitting the model to experimental data.

The parametrization is generally non-unique: different sets of parameters can be used to represent the same data. This makes this procedure somehow arbitrary, except for the fact that the number of parameters should at least equal the dimensionality of the system.

Parametrization is in general not directly related to the concept of multi-level and hybrid models as a whole. It is rather a problem related to the different models composing the multi-level description. Nevertheless, the effective number of parameters of a model is a good measure of its complexity [40], which, in case of multi-level models, is usually very high. Parametrization therefore requires the development of new methods able to handle the complexity of the system. This is further exacerbated by the fact that, in multi-level models, quite often parameters of a given layer represent variables for an upward layer [12]. It is therefore important to review common practices to perform this task.

In [41], the authors present a platform named ABC-SysBio which provides tools for parameter estimation and model selection in systems biology. Parameter estimation is performed with approximate Bayesian computation [42]. ABC-SysBio is designed to work with models written in SBML. Deterministic and stochastic models can be analyzed in ABC-SysBio. A criticality of this approach is the fact that it is computationally expensive. On the other hand, it provides a high-level of detail on the system to be modelled.

Bayesian numerical techniques are pretty effective for inferring the parameter values of complex models, in particular when ODEs are used as formalism, which is often the case in systems biology studies. In [43], the authors present GNU MCSim, a numerical simulation tool able to perform Bayesian statistical inference for algebraic or differential equation systems. This tool supports different kinds of simulations, including simple runs, as well as plain or Markov Chain Monte Carlo simulations [44]. Finally, with an optimal design procedure the tool optimizes the number and location of observation times for different experimental conditions, while minimizing parameter and output variance for a given model.

Besides relations between the parts of a biological system, parameters can also refer to other aspects in process evolution, such as time-delays in regulatory networks. The mechanisms causing them are often unknown and probably multi-factorial, making the task of parameter identification for a model comprising them an ill-defined problem. In [45], the authors present a semi-parametric hybrid approach for performing system identification for biochemical networks with time-delays, obtaining significantly better prediction performances than models overlooking them.

The complexity of a model corresponds to a wide set of possible trajectories in the system evolution. This can make the search for good parameters a computationally intensive task. In [46], the authors present Breach, a Matlab/C++ toolbox for verification and parameter synthesis for hybrid non-linear models. This approach is based on a very efficient numerical solver of ODEs that is able to handle the complexity of the task. Parameters synthesis is property-driven and based on Signal Temporal Logic [47].

As already mentioned, parametrization is non-unique: different model structures can generate accurate descriptions of the same system. Similarly, parameter identification as well can yield non-unique results, as underlined in [48]. In this work, the authors empirically tested 17 systems biology models from the literature, examining how sensitive their behaviour was to changes in the value of the parameters. They find that all models under analysis have loosely constrained (or sloppy) parameter sensitivities, and claim that this sloppiness is universal in systems biology models. Authors underline that sloppiness is an intrinsic aspect of any biological system, and should not be considered as a failure of the model. Of course, inaccuracies in the experimental procedures are sources of inaccuracies in the model; nevertheless, another possible explanation is that the effects of different parameter combinations on the behaviour of the system may be redundant. Overall, this work highlights the critical aspect of parameter uncertainty in systems biology models. It suggests that, since parametrized models universally exhibit such sloppiness in the parameters' values, models should be intended less as reliable knowledge bases ([49] provides a good example of that) describing quantitative relations between system components by means of parameters, and more as tools for making as accurate as possible predictions on the behaviour of the system [50].

### 3.4 Verification and validation

Verification is the process of finding and fixing model errors, assuring that the model matches the starting assumptions and specifications [51]. In other words, verification ensures model correctness.

As anticipated in Section 2 and Subsection 3.3, hybrid and multi-level models result from the integration of other existing models, which in general already passed through separate verification processes. However, for such composite models, verification concerns also the way models communicate between different levels and formalisms [52].

All these aspects must be considered when performing verification on hybrid and multi-level models, and the development of dedicated tools is auspicable. In [53], the authors present UPPAAL, an integrated tool environment taking care of model construction, validation and verification of dynamical hierarchical hybrid systems. UPPAAL consists of three main parts: a description language, a non-deterministic guarded command language supporting multiple data types; the simulator, allowing for validation through examination of *possible* dynamic executions of a system during early phases of the design process; a model-checker, performing verification of the model by *exhaustive* exploration of the entire state-space of the system. UPPAAL makes an exemplary tool environment since it combines the efficiency due to functional integration of different tools covering the entire modelling process with being easy to use. In particular, the validation approach, performed early in the design phase, allows for adjusting the model construction process in a guided way, saving time during the following phases.

Validation is the process of making sure that the model represents the system to be modelled at a sufficient level of accuracy [51]. Techniques such as cross-validation assess to what degree the model under investigation generalizes to a data set not used for the model construction.

When performing validation on hierarchical Bayesian models in phylogenetics, the most common approach is to investigate marginal likelihoods [54]. But, as noted in [55], this approach is very sensitive to the model priors. For avoiding this issue, the authors present an alternative approach based on the expansion of the cross-validation method proposed in [56], to include other components of the Bayesian hierarchical model in the rotation estimation process.

Debugging, verification and validation of a model often undergo many iterations. Still, it is necessary to keep in mind that models are abstract representations of a system, producing approximations of its behaviour: verification and validation processes are not intended to aim at maximum accuracy, but rather at an arbitrarily defined “satisfactory level”.

## 4 Model selection: trading-off computational complexity of simulation and accuracy

As introduced in Subsection 3.4, a necessary premise to keep in mind when approaching the modelling process is that “all models are wrong” [57]. They are abstractions of a system or process of interest, designed to get a better insight over a given phenomenon. Many arbitrary choices must be taken during the modelling activity, resulting in a number of different models describing the system of interest in a seemingly equivalent way. The model selection task is the process of picking the best model among them. For a good review of the existing approaches to model selection in systems and synthetic biology the reader may refer to [58]. Anyhow, the question is how to define suitable evaluation criteria for making this choice.

The complexity of systems biology models usually pairs with non-linearity and stochasticity. To be predictive of the system behaviour, a model must be able to reproduce the dynamical evolution of the corresponding processes. This in turn requires the ability to simulate the model, translating the biological complexity in a computational problem. As stated in Subsection 3.3, a measure for a model's complexity is the effective number of parameters [40]. On average, models in systems biology aim at representing large networks of interacting entities, each interaction corresponding to one of such parameters. In the case of multi-level models, this virtually holds for all levels composing the model and for all communication channels between levels. More in general, compared to single level models, hierarchical and hybrid models intrinsically try to represent to a larger extent the complexity of the real system in order to obtain higher model accuracy. This comes at the cost of larger computational complexity during simulations.

A good systems biology model should carefully trade-off accuracy and complexity. This is usually obtained through the application of complexity reduction algorithms [59]. These algorithms are able not only to speed up the simulations, but also to help split the system in smaller subsystems that can be studied independently, thus improving the understanding of the system under study [60].

Another way to reduce the cost of the computation when dealing with massive amounts of data coming from a highly structured hierarchical system is to orient the scope of the modelling process in a narrow way. This is the strategy chosen in [36], where the complexity of modelling an organ (virtual liver) accounting for all phenomena from the molecular to the organismal scale is clearly prohibitive. The method applied to tackle this complexity has been to narrow the whole modelling process to the simulation of specific liver functionalities or diseases. This allowed for the selection of model components improving the accuracy when studying the selected functionalities and diseases without including other details not strictly related to the target scope of the research.

When simulating hybrid models, another important aspect must be taken into account: simulation engines should be able to handle multiple formalisms concurrently. This is the case of Flint, the tool used in [33] for simulating hybrid SBML-PHML models. More specifically, Flint extracts abstract syntax trees (ASTs) from the SBML model using the SBML ODE solver SOSlib [61]. After that, while preserving model consistency, Flint merges ASTs into formulas from the PHML model, and from these generates the bytecode for executing a simulation.

Maximization of accuracy is another top priority: the model must represent the system as precisely as possible given the availability of enabling prior information and data. This reflects in the model's capability to correctly predict the system behaviour, accepting a certain degree of uncertainty [62]. Predictions can for instance be made about future system evolution, or system behaviour under different conditions.

Maximizing the model accuracy may also have the objective of improving the reliability of information held in the model. In fact, in systems biology, models are intended as very informative knowledge bases as well [2], [49].

The aftermath of accuracy maximization is often the extensive inclusion of biological complexity in the model. This yields high computational complexity, which is a problem because it can possibly lead to unacceptable computational times. One way for approaching this problem is by incrementing the computational power, for instance by running simulations on high-performance distributed computing systems, as in [36].

On the theoretical side instead, the general strategy is to reduce model complexity while preserving model accuracy. Complexity reduction can be achieved introducing simplifications in the model in a way to preserve the accuracy on the most relevant portions of the system. An example of this approach is proposed in [24], where parametrization of a complex metabolic network results in detailed mechanistic equations when representing crucial mechanisms in the system, and in simplified representations when referring to less relevant ones.

For model selection, a leading principle in this sense is the use of an approach inspired by the Ockham's razor principle: given equivalent performance (accuracy), the simplest (least complex) model is always the best one. This way of reasoning also goes under the name of the principle of parsimony [63], [64].

When trying to balance these aspects, for making appropriate choices it is important to keep in mind the objectives of the specific modelling strategy. If the model is for instance intended as a tool to perform accurate predictions over system behaviours(as it should be according to [48]), the trade-off between maximizing accuracy and respecting the principle of parsimony is the more relevant constraint. In this perspective, other issues concerning parameter uncertainty and model understandability are to be considered less urgent as long as predictions are accurate and computationally feasible in suitable time.

Conversely, if the scope of the modelling process is to provide a reliable and understandable knowledge base for a biological system, the modelling process should focus on other human-related aspects such as clarity and understandability [65].

Maximization of accuracy should also target the single-parameter values, making each part of the model re-usable in the future for other models, and by other modellers. In many situations, parameters optimization should not overlook the re-usability of the selected model in future problems, and by other researchers. In other words, a leading principle for model selection in this scenario is the enhancement of model re-usability [66] and interoperability [67]. This reflects the necessity that exists in systems biology of efficiently and reliably sharing validated and structured information. This can be achieved by valuing and sharing contributions from different domains of expertise and professional figures involved in the process. In fact, efforts for advancing a multifaceted domain such as systems biology require a collaborative contribution by experimentalists and theoreticians, scientists and engineers [2], [68], [69]. Every counterpart possibly works producing information from a different system level, and comes from a different knowledge domain, with its peculiar history and perspective, which reflects in the way information translates into knowledge.

A good knowledge base for supporting such heterogeneous community-based contribution to systems biology must then handle information which both comes from different system levels and is specified using different formalisms. That is exactly what models which are both multi-level and hybrid do, and what makes them valuable tools for getting better insights over biological complexity while valuing and expanding the existing knowledge of biological systems in general.
